# Pembrolizumab-Induced Pancreatitis: Take It With a Grain of Salt

**DOI:** 10.7759/cureus.58417

**Published:** 2024-04-16

**Authors:** Prachi C Gajjar, Parth Parmar, Hetvi Gajjar, Shikha Upreti, Munir Shah

**Affiliations:** 1 Internal Medicine, Western Reserve Health Education, Warren, USA; 2 Internal Medicine, Pramukhswami Medical College - Karamsad, Karamsad, IND; 3 Internal Medicine, Ross University School of Medicine, Miami, USA; 4 Infectious Disease, Western Reserve Health Education, Warren, USA

**Keywords:** castrate-resistant metastatic prostate cancer, prostate cancer, programmed cell death protein (pd-1), immunomodulator, pancreatitis, pembrolizumab

## Abstract

Keytruda (pembrolizumab) is an immunomodulator that prevents the interaction between programmed cell death protein (PD-1) and programmed death ligand (PD-L1/2) on immune cells and tumour cells, thereby preventing T cell dysfunction. At times, mounting a strong immune response against tumour cells may not spare normal cells, leading to a variety of multisystemic adverse effects. With this, we present a case of a 64-year-old male who developed acute pancreatitis after completing eight cycles of Keytruda for castrate-resistant metastatic prostate cancer for six months, after all other causes of pancreatitis were excluded.

## Introduction

Immune checkpoints consist of certain receptor proteins such as programmed cell death protein 1 (PD-1) and cytotoxic T-lymphocyte-associated protein 4 (CTLA-4) on immune cells, like T cells, B cells, and natural killer cells. These receptor proteins act as an “off switch," preventing a strong autoimmunity in the body. On the other hand, their counterpart receptor, protein programmed death-ligand 1 (PDL-1/2), either present on normal cells or tumour cells binds with the PD-1 receptor on immune cells to neutralize the intrinsic immunity, which otherwise would fight back the tumour cells. The discovery of novel immunotherapy agents, known as immune checkpoint inhibitors (ICIs), was considered groundbreaking, as they prevent interaction between PD-1 and PD-L1/2, thus facilitating a strong immune response against the tumour cells. At this time, the U.S. Food and Drug Administration has approved three different types of ICIs: PD-1 inhibitors (nivolumab, pembrolizumab, and cemiplimab), PDL-1 inhibitors (atezolizumab, durvalumab, and avelumab), and CTLA-4 inhibitor (ipilimumab) [1,2].

## Case presentation

A 64-year-old male with an established history of cholecystectomy, castrate-resistant metastatic prostate cancer, status post-cyst-prostatectomy with exoneration, proctosigmoidectomy and ileal conduit with a urinary diversion in 2015, and metastatic pulmonary nodules with biopsy suggestive of high microsatellite instability (MSI-H) adenocarcinoma likely from a colorectal source presented to the emergency department with severe abdominal pain localised to the upper abdomen, nausea, and four episodes of non-projectile, non-bilious vomiting.

The patient had an insignificant social history as he was non-alcoholic and a non-smoker, and he never used recreational drugs. At the time of admission, he was diagnosed with pancreatitis based on the significant elevation of his pancreatic enzyme laboratory values, with lipase and amylase values of 800 U/L and 700 U/L, respectively. In addition, the patient's lipid panel, including a serum triglyceride level and IgG4 antibody levels, was unremarkable. These pancreatic enzyme values continued to uptrend for two days before downtrending on day three.

**Figure 1:**
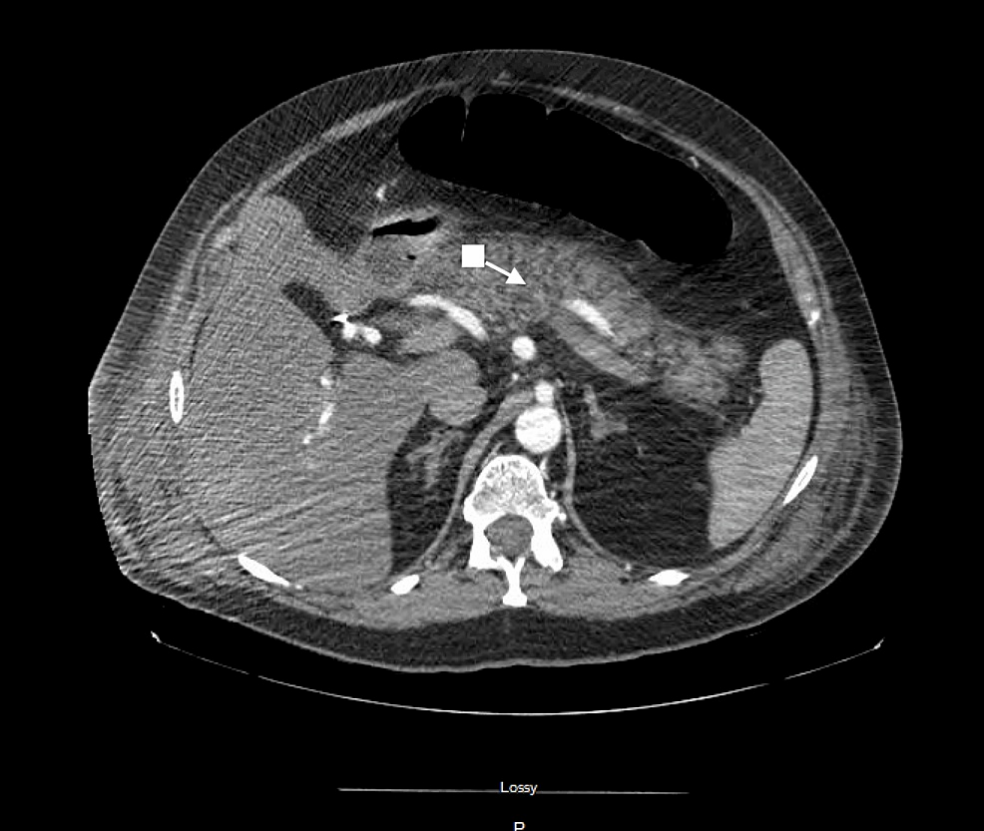
CT showing inflamed pancreas (arrow) CT: computed tomography

Abdominal imaging with a contrast-enhanced CT scan demonstrated duodenitis, fatty infiltrate of the liver, right-sided ostomy, and hernia without bowel incarceration located at the midline of the anterior abdominal wall (Figure 1).

**Figure 2:**
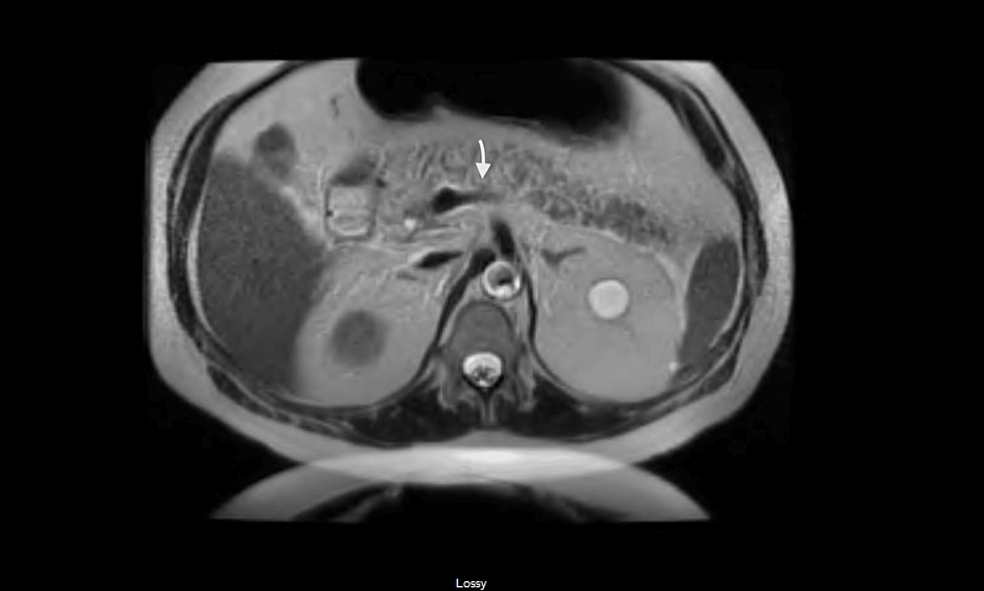
Magnetic resonance cholangiopancreatography showing normal calibre bile ducts without intraluminal filling defects (arrow)

Further imaging with magnetic resonance cholangiopancreatography displayed wall thickening in the region of the duodenum with mild peripancreatic fat stranding and fluid. These results confirmed the finding of acute pancreatitis and duodenitis and ruled out pancreatic necrosis or loculated collection. In addition, post-cholecystectomy changes with normal calibre bile ducts without intraluminal filling defects were identified (Figure 2).

Of relevance, the patient was started on pembrolizumab (Keytruda) every 21 days for the last six months for his metastatic lung cancer as he had a deficient mismatch repair (dMMR). For prostate cancer, he was on androgen deprivation therapy such as enzalutamide and denosumab for the last six years.

As the most common causes of pancreatitis, such as alcoholism, gallstones, sphincter of Oddi dysfunction, and hypertriglyceridemia, were ruled out based on history, lab values, and imaging, the most likely aetiology of the patient's pancreatitis was narrowed down to his newly added immunotherapy, pembrolizumab. The Naranjo adverse drug reaction probability score for our patient was 6, making it a probable adverse drug reaction related to pembrolizumab.

## Discussion

Tumour cells express certain proteins called PD-L1/2, which interact with PD-1 proteins on immune cells like T cells, B cells, and natural killer cells, leading to immune cell dysfunction. T cell exhaustion leads to the further proliferation of cancer cells. PD-1 monoclonal antibodies like pembrolizumab directly inhibit PD-1 receptors on T cells, thereby strengthening immune attacks on tumour cells. Keytruda (pembrolizumab) is an immunomodulator that is approved by the U.S. Food and Drug Administration for the treatment of solid tumours with positive MSI-H or dMMR genes.

However, this widespread activation of T-lymphocytes as it starts attacking non-tumour cells is associated with various immune-related adverse events affecting multiple systems, such as the gastrointestinal, endocrine, dermatological, nervous system, and musculoskeletal systems. The most common side effects include rash, diarrhoea, pneumonitis, hepatitis, and nephritis. Pancreatitis can occur in rare instances.

Pancreatic injuries are not commonly associated with these immune checkpoint inhibitors (ICIs). Most of the pancreatic adverse events, such as pancreatitis, asymptomatic pancreatic enzyme elevation, hypoglycemia, diabetes mellitus, and exocrine pancreatic insufficiencies, are only reported as case reports. The mechanism of action can be briefly explained by T-lymphocytes densely infiltrating in and around the pancreatic islets, destroying exocrine and endocrine pancreatic tissues [1]. Ashfaq et al. described a case of pembrolizumab-induced hypertriglyceridemia pancreatitis with a possible formation of autoantibodies against glycosylphosphatidylinositol-anchored high-density lipoprotein binding protein 1 by the novel medicine [3].

Diagnosing ICI-induced pancreatitis can be challenging due to the duration of the onset of pancreatitis, and the initiation of the culprit drug is variable and unpredictable. Our patient developed pancreatitis after eight cycles (six months) of pembrolizumab, whereas a wide range of duration and onset from three to 51 weeks has been described in the literature [1].

The clinical presentation of ICI-induced pancreatitis is usually similar to that of normal pancreatitis, requiring the presence of two of the following three clinical features: (1) epigastric pain radiating to the back; (2) elevated pancreatic enzymes lipase/amylase at least three times the upper normal limit; and (3) a characteristic finding of pancreatitis on abdominal imaging. A meta-analysis recorded an incidence of asymptomatic elevation of pancreatic enzymes in 2.7% of cases and acute pancreatitis in 1.9% of cases [2]. However, National Comprehensive Cancer Network guidelines do not warrant holding ICI treatment with symptomatic pancreatic enzyme elevation up to three times the upper normal limit or without overt pancreatitis [4].

ICI-induced pancreatitis is managed similarly to any other acute pancreatitis with hydration. Additionally, it is recommended to discontinue ICIs along with initiating tapering doses of steroids (0.5-1 mg/kg) only if needed. In our case, the patient did not require steroids. In contrast, five out of seven patients in a case series by Hana et al. Al required steroids for resolution [5].

## Conclusions

It has been a decade since ICIs such as pembrolizumab were discovered. While ICIs have become widely used in today's medical practice, further research and trials are necessary to refine the medication’s safety profile. Our case report adds to the limited literature available on this subject, emphasising the need for a thorough investigation.
